# Redefining chemotherapy-induced peripheral neuropathy through symptom cluster analysis and patient-reported outcome data over time

**DOI:** 10.1186/s12885-019-6352-3

**Published:** 2019-11-27

**Authors:** Mian Wang, Hui Lin Cheng, Violeta Lopez, Raghav Sundar, Janelle Yorke, Alex Molassiotis

**Affiliations:** 1 School of Nursing, The Hong Kong Polytechnic University, Hong Kong, Hong Kong SAR; 2 Alice Lee Centre for Nursing Studies, National University of Singapore, Singapore, Singapore; 3 The N.1 Institute of Health, National University of Singapore, Singapore, Singapore; 4 Division of Nursing, Midwifery & Social Work, University of Manchester, Manchester, UK; 5 Christie Patient Centred Research (CPCR), The Christie NHS Foundation Trust, Manchester, UK

**Keywords:** Cancer, Chemotherapy-induced peripheral neuropathy, Patient-reported outcome, Symptom clusters

## Abstract

**Background:**

Chemotherapy-induced peripheral neuropathy (CIPN) is common among cancer patients treated with neurotoxic chemotherapy agents. Better knowledge on symptom clusters of CIPN may help improve symptom management in clinical practice. This study aimed to identify symptom clusters of CIPN and to map their trajectories before initiation of chemotherapy to 12-month follow-up.

**Methods:**

A secondary analysis of a longitudinal dataset was conducted using principal component approach. The European Organization for the Research and Treatment of Cancer Quality of Life Questionnaires Core 30 and CIPN 20 were used to measure symptom clusters of CIPN in patients with mixed cancer diagnosis across 10 time points over 12 months.

**Results:**

Sample size in each assessment point ranged from 118 to 343 participants. Four CIPN symptom clusters were identified, including a clear sensory neuropathy symptom cluster, a mixed motor-sensory neuropathy symptom cluster, a mixed sensorimotor neuropathy symptom cluster, and a less clear autonomic neuropathy symptom cluster. The core symptoms in each symptom cluster were mostly stable while the secondary symptoms changed over time.

**Conclusions:**

The analysis suggests that CIPN is predominantly a sensory neuropathy with no evidence of a pure motor dysfunction but with mixed motor-related and autonomic changes accompanying sensory dysfunctions over time. Future symptom management strategies can be designed based on the morphology of CIPN.

## Background

Chemotherapy-induced peripheral neuropathy (CIPN) is a common side effect in cancer patients treated with neurotoxic agents [1]. Symptoms of CIPN are diverse and have been classified as three major dimensions including sensory, motor, and autonomic, with sensory symptoms being predominant [1–3]. These symptoms often exist simultaneously and affect cancer patients by causing paresthesia, impairing function and damaging hearing and vision, etc. [4, 5]. Studies also demonstrate that CIPN can lead to psychological issues like anxiety, depression, and stress disorder [6, 7]. Both the physiological and psychological symptoms are known to decrease cancer patients’ quality of life [8]. Severe CIPN may force patients to prematurely discontinue chemotherapy, which would reduce anticancer treatment effects and possibly decrease overall survival [9].

Given the multidimensional and interrelated features of CIPN symptoms, research on symptom clusters may help improve our understanding of CIPN symptoms and develop appropriate strategies for symptom management. A symptom cluster refers to a group of two or more correlated and concurrent symptoms experienced by patients [10]. Additionally, it should include at least one core symptom that is consistent overtime [11]. Although symptom clusters research in oncology has developed for nearly two decades, the symptom clusters of CIPN are not fully understood. Few studies identified certain symptom clusters related to CIPN including numbness/tingling in hands/feet, feeling drowsy, and pain [11–13]; however, these findings were not consistent due to either a short duration of observation or the use of generic but not CIPN-specific measurements. A recent literature review identified 19 studies investigating chemotherapy-related symptom clusters, but none of them clearly mapped symptom clusters of CIPN [14]. Considering the differences between CIPN and general side-effects of chemotherapy, it is essential to identify the nature of CIPN-specific symptom clusters. This study aimed to explore the morphology and the patterns of CIPN symptom clusters in cancer patients throughout the course of chemotherapy and up to 12 months.

## Methods

### Design

This is a secondary analysis of data from a longitudinal study, which aimed to examine the prevalence, risk factors, and patterns of CIPN in cancer patients [15]. The study was approved by the ethical review committee of the universities and hospitals involved. To identify the patterns and changes of CIPN symptom clusters over time, data were collected from baseline (T1) through six cycles of chemotherapy (T2-T7) and at six- (T8), nine- (T9), and 12-month (T10) follow-up.

### Sample and settings

A total of 343 patients were recruited from three hospitals in Hong Kong, United Kingdom, and Singapore. The inclusion criteria were the patients 1) aged ≥18 years; 2) were diagnosed with cancer; 3) were beginning to receive neurotoxic chemotherapy; and 4) were anticipated to have a life expectancy ≥12 months as determined by oncologists.

### Measures

#### European Organization for the Research and Treatment of Cancer Quality of Life Questionnaire-Core 30 (EORTC QLQ-C30)

The EORTC QLQ-C30 is a widely used self-reported core questionnaire measuring health-related quality-of-life (QoL) in cancer patients [16]. It includes a total of 30 items assessing cancer patients’ functions, symptoms, financial issue, and overall health status and QoL. Previous studies have demonstrated good psychometric properties of both the English and Chinese versions of the EORTC QLQ-C30 [2, 17, 18]. In the present study, items were selected based on the description of chemotherapy-related symptom clusters by Yates et al. [12], including dyspnea, pain, fatigue, insomnia, appetite loss, nausea and vomiting, constipation, diarrhea, cognitive function, and emotional function.

#### EORTC QLQ-CIPN20

The EORTC QLQ-CIPN20 is an additional module to the core EORTC-QLQ-C30 questionnaire with 20 items assessing sensory, motor, and autonomic symptoms experienced by patients during the past week. Each item can be scored from 1 (not at all) to 4 (very much), with higher scores indicating worse symptom severity. Both the English and Chinese versions of the EORTC QLQ-CIPN20 were reported to have good stability, reliability, validity, and responsiveness to change [2, 4, 19]. As the item 19 “Did you have difficulty using the pedals?” and item 20 “Did you have difficulty getting or maintaining an erection?” were not applicable to most of the patients included in the sample and caused large missing data, only items 1–18 were used in this study.

### Statistical analysis

Data analysis was performed using SPSS version 25.0 (IBM, Inc., Chicago, IL). As this is an exploratory study, a principal component analysis (PCA) was used to identify potentially clustered symptoms of CIPN. Similar to previous studies, the method of varimax rotation was adopted to determine the distribution of symptoms without over- or under-estimating their relationship [20]. The PCA with varimax rotation was performed on the data at each time point and then compared over time. The Kaiser-Meyer-Olkin (KMO) measure (cut-off value > 0.50) and the Bartlett’s test of Sphericity (*P* < 0.05) were performed to evaluate adequacy of sample size and suitability for the analysis.

The components (i.e. symptom clusters) containing at least one consistent EORTC QLQ-CIPN20 item (i.e. symptom associated with CIPN) over time and having eigenvalue > 1.0 were extracted. Internal consistency of each identified component was determined by Cronbach’s α. Since this is an exploratory study and there is no specific rule to determine the strength of relationships between symptoms in a cluster [21], no specific cut-off value was set for loading of symptoms (although it is acknowledged that any clusters with loadings of < 0.50 should be treated cautiously [21]). Only symptom items that were considered to be both statistically and clinically meaningful were retained in relevant symptom clusters, considering that both aspects are important [21]. Symptom items cross-loaded in more than one cluster were accepted and were used to identify latent correlations between symptom clusters when considered as clinically meaningful.

To further confirm the CIPN symptom clusters, PCA with varimax rotation were conducted with docetaxel, paclitaxel and carboplatin plus paclitaxel, as well as cisplatin and carboplatin subgroups. Multivariate linear regression was used to explore the potential influence of patients’ demographic and clinical characteristics on composite score of CIPN symptom clusters.

## Results

### Sample characteristics

In this study, sample size at each assessment time point ranged from 118 to 343 due to chemotherapy cessation, death, or relocation of patients. Age of the patients ranged from 33 to 79 years, with an average age of 55.15 years. Majority of the sample was female (*n* = 256, 74.6%). Detailed demographic and clinical characteristics of the patients are listed in Table 1.

**Table 1:** Sample characteristics (*n* = 343)

Characteristics	Mean ± SD / n (%)
Age (years)	55.15 ± 9.36
Race	
Chinese	269 (78.4)
Non-Chinese Asians	31 (9.0)
Caucasian	43 (12.5)
Gender	
Female	256 (74.6)
Male	87 (25.4)
Cancer diagnosis	
Gynecological (ovary, cervix, endometrium, genital tract)	45 (13.1)
Lung	48 (14.0)
Head and neck	30 (8.7)
Breast	174 (50.7)
Urinary tract (prostate, bladder, uterus)	17 (5.0)
Gastrointestinal (esophagus, pancreas, stomach, bile duct, colon-rectum)	29 (8.5)
Cancer stage	
Stage I	52 (15.2)
Stage II	99 (28.9)
Stage III	116 (33.8)
Stage IV	76 (22.2)
Treatment intent	
Radical (adjuvant)	199 (58.0)
Radical (neoadjuvant)	51 (14.9)
Radical (concurrent)	30 (8.7)
Palliative	63 (18.4)
Chemotherapy agents	
Docetaxel	122 (35.6)
Paclitaxel	33 (9.6)
Cisplatin	42 (12.2)
Carboplatin	8 (2.3)
Oxaliplatin	9 (2.6)
Carboplatin + Paclitaxel	50 (14.6)
Carboplatin + Docetaxel	29 (8.5)
Vinorelbine	31 (9.0)
Xelox	19 (5.5)
Diabetes History	
Yes	54 (15.7)

*Abbreviation*: *SD* standard deviation

### Symptom clusters of CIPN over time

Four symptom clusters of CIPN were identified across all the assessments before initiation of chemotherapy to 12-month follow-up after completion of chemotherapy. These symptom clusters were defined as the sensory neuropathy symptom cluster, the motor-sensory neuropathy symptom cluster, the sensorimotor neuropathy symptom cluster, and the autonomic neuropathy symptom cluster. Despite the change of sample size over time, the data was suitable for principal component analysis at all of the assessment time points with the KMO values ranging from 0.74 to 0.89 and the Bartlett’s test of Sphericity remaining statistically significant (*P* < 0.001).

### The sensory neuropathy symptom cluster

The clear sensory neuropathy symptom cluster was identified with three consistent core symptoms (i.e., tingling in the feet, tingling in the hands, and numbness in the feet). Burning pain in the hands was a secondary symptom at the first two time points, but was then replaced by numbness in the hands in the rest of the time points. Cramps in the hands/feet presented in this symptom cluster at the first three time points and then was more prominent in the sensorimotor symptom cluster until the last assessment time point, whereas cramps in the feet reappeared in the sensory neuropathy symptom cluster. From the T4 assessment onwards, the sensory neuropathy symptom cluster contained the three core symptoms and one secondary symptom of numbness in the hands. Although the two symptoms, numbness in the feet and numbness in the hands, were cross-loaded in another cluster with general symptoms (i.e., having difficulty remembering things) at T7, the structure of the symptom cluster remained stable until the T9 assessment, where the symptom cluster included only the three core symptoms. Vomiting and nausea appeared in this symptom cluster at T10. Despite the dynamic structure, internal consistency of the sensory neuropathy symptom cluster remained high over time (Cronbach’s α ranged between 0.82 and 0.93) (Table 2).

**Table 2:** Symptom clusters of chemotherapy-induced peripheral neuropathy over time

Symptom Cluster	Symptoms	T1	T2	T3	T4	T5	T6	T7	T8	T9	T10
The sensory neuropathy symptom cluster	**Tingling feet**	**•**	**•**	**•**	**•**	**•**	**•**	**•**	**•**	**•**	**•**
	**Tingling hands**	**•**	**•**	**•**	**•**	**•**	**•**	**•**	**•**	**•**	**•**
	**Numbness in feet**	**•**	**•**	**•**	**•**	**•**	**•**	**•**	**•**	**•**	**•**
	Numbness in hands			**•**	**•**	**•**	**•**	**•**	**•**		**•**
	Burning pain in hands	**•**	**•**								
	Burning pain in feet	**•**	**•**								
	Cramps in hands	**•**	**•**								
	Cramps in feet		**•**	**•**							**•**
	Vomiting										**•**
	Nausea										**•**
	Loadings	0.47–0.88	0.51–0.76	0.54–0.87	0.70–0.85	0.77–0.82	0.55–0.84	0.50–0.90	0.65–0.84	0.66–0.84	0.53–0.81
	Internal consistency	α = 0.86	α = 0.86	α = 0.86	α = 0.91	α = 0.88	α = 0.82	α = 0.93	α = 0.87	α = 0.82	α = 0.87
	Variance explained (%)	11.62	12.37	12.42	9.56	9.57	7.87	7.41	9.01	7.29	15.96
The motor-sensory neuropathy symptom cluster	**Manipulating small objects**	**•**	**•**	**•**	**•**	**•**	**•**	**•**	**•**	**•**	**•**
	**Holding pen**	**•**	**•**	**•**	**•**	**•**	**•**	**•**	**•**	**•**	**•**
	Opening jars or bottles	**•**			**•**				**•**		
	Difficulty walking because feet dropped downwards	**•**		**•**					**•**		
	Climbing stairs			**•**					**•**		
	Cramps in hands			**•**							
	Distinguishing hot-cold				**•**	**•**	**•**	**•**			
	Burning pain in hands					**•**		**•**			
	Burning pain in feet							**•**			
	Standing/walking from difficulty feeling the ground under feet								**•**		
	Dizzyness								**•**		
	Loadings	0.49–0.78	0.65–0.65	0.46–0.77	0.50–0.78	0.49–0.79	0.81–0.82	0.55–0.71	0.44–0.79	0.81–0.86	0.65–0.84
	Internal consistency	α = 0.58	α = 0.62	α = 0.81	α = 0.77	α = 0.78	α = 0.86	α = 0.76	α = 0.87	α = 0.85	α = 0.64
	Variance explained (%)	7.46	5.36	8.96	6.90	7.58	9.36	8.48	10.10	6.33	5.39
The sensorimotor neuropathy symptom cluster	**Standing/walking from difficulty feeling the ground under feet**	**•**	**•**	**•**	**•**	**•**	**•**	**•**	**•**	**•**	**•**
	Difficulty remembering	**•**									
	Difficulty with concentration	**•**									
	Difficulty walking because feet dropped downwards		**•**		**•**	**•**	**•**	**•**	**•**	**•**	**•**
	Distinguishing hot-cold		**•**	**•**							
	Climbing stairs		**•**		**•**		**•**			**•**	**•**
	Burning pain in hands			**•**	**•**						
	Burning pain in feet			**•**	**•**						
	Cramps in hands				**•**		**•**	**•**	**•**		**•**
	Cramps in feet				**•**		**•**	**•**			
	Nausea								**•**		
	Weakness in legs								**•**		
	Constipation								**•**		
	Needs resting								**•**		
	Tired								**•**		
	Having trouble sleeping								**•**		
	Pain									**•**	
	Opening jars or bottles										**•**
	Loadings	0.80–0.91	0.45–0.77	0.37–0.69	0.54–0.72	0.78–0.84	0.52–0.72	0.22–0.82	0.22–0.70	0.39–0.81	0.63–0.88
	Internal consistency	α = 0.82	α = 0.72	α = 0.80	α = 0.86	α = 0.81	α = 0.81	α = 0.70	α = 0.86	α = 0.84	α = 0.88
	Variance explained (%)	7.58	7.19	5.79	12.09	6.19	8.54	5.29	13.61	10.61	11.01
The autonomic neuropathy symptom cluster	**Blurred vision**	**•**	**•**	**•**	**•**	**•**	**•**	**•**	**•**	**•**	**•**
	Climbing stairs	**•**						**•**			
	Dizzyness	**•**	**•**					**•**	**•**		**•**
	Difficulty with concentration		**•**		**•**				**•**	**•**	
	Difficulty remembering		**•**						**•**	**•**	
	Pain		**•**					**•**			
	Needs resting		**•**								
	Having trouble sleeping		**•**								
	Constipation		**•**								
	Shortness of breath		**•**								
	Opening jars or bottles		**•**								
	Difficulty hearing			**•**	**•**	**•**			**•**	**•**	
	Burning pain in feet					**•**	**•**				
	Burning pain in hands						**•**				
	Standing/walking from difficulty feeling the ground under feet							**•**			
	Difficulty walking because feet dropped downwards							**•**			
	Loadings	0.86–0.97	0.42–0.68	0.69–0.81	0.47–0.87	0.44–0.70	0.41–0.77	0.47–0.61	0.40–0.72	0.57–0.68	1.00–1.00
	Internal consistency	α = 0.90	α = 0.78	α = 0.57	α = 0.63	α = 0.59	α = 0.58	α = 0.78	α = 0.67	α = 0.66	α = 1.00
	Variance explained (%)	7.56	10.49	5.66	5.43	5.58	5.72	6.96	6.51	6.56	5.80

Bold is used to indicate core symptom(s)

### The motor-sensory neuropathy symptom cluster

The mixed motor-sensory neuropathy symptom cluster with primarily motor symptoms was identified with two core symptoms (i.e., having difficulty manipulating small objects and having a problem holding a pen). The majority of secondary symptoms in this cluster were motor neuropathy symptoms including having difficulty opening a jar, having difficulty walking because feet dropped downward, and having difficulty climbing stairs. Cramps in the hands presented in this symptom cluster at time point (T3). Few sensory neuropathy symptoms, including having difficulty distinguishing between hot and cold water, burning pain in the hands/feet, and having problems standing or walking because of difficulty feeling the ground under the feet, were also identified in this symptom cluster from T4 to T8. Internal consistencies of the motor-sensory neuropathy symptom cluster were acceptable from T3 to T9 assessments (Cronbach’s α ranged from 0.76 to 0.87), but were low at T1 and T2 (Cronbach’s α were 0.58 and 0.62, respectively) and T10 (Cronbach’s α = 0.64) assessments (Table 2).

### The sensorimotor neuropathy symptom cluster

The sensorimotor neuropathy symptom cluster, another mixed cluster but with primarily sensory symptoms this time, was identified with a single core symptom, namely having problems standing or walking because of difficulty feeling the ground under the feet. Such core symptom was consistent from T1 to T6 as well as at T9 and T10 with its loading ranging between 0.55 and 0.88 across these time points. However, it was only cross-loaded in the cluster at T7 and T8 with small loadings of 0.22 and 0.29, respectively. In terms of secondary symptoms, both sensory and motor neuropathy symptoms were included in the sensorimotor neuropathy symptom cluster over time. Cognitive symptoms including having difficulty remembering things and having difficulty in concentration were found at T1. Several general symptoms pertaining to motor and autonomic changes were identified at T8 and T9 including weakness, needing rest, being tired, having trouble sleeping, or experiencing nausea, constipation, and pain. Although cross-loaded core and secondary symptoms were identified at two assessment time points (T7 and T8), internal consistency of the symptom cluster was acceptable over time (Cronbach’s α ranged between 0.70 and 0.88) (Table 2).

### The autonomic neuropathy symptom cluster

The autonomic neuropathy symptom cluster was identified with the core symptom of blurred vision. Dizziness, having difficulty hearing, having difficulty in concentration, and having difficulty in remembering things were the most common secondary symptoms in this symptom cluster over time. Few other sensory (i.e. burning pain in hands/feet and having problems standing or walking because of difficulty feeling the ground under the feet), motor (i.e. having difficulty climbing stairs, having difficulty opening a jar, and having difficulty walking because the feet dropped downward), and general symptoms (i.e. pain, needing rest, trouble sleeping, constipation, and short breath) also presented in this symptom cluster at half of the assessment time points. However, the autonomic neuropathy symptom cluster was not stable as its internal consistency coefficient was acceptable at only four (T1, T2, T7, and T10) out of the ten assessment time points (Cronbach’s α = 0.90, 0.78, 0.78, and 1.00, respectively) (Table 2).

### Subgroups and influence factors analyses

A subgroup analysis was performed to verify the overall results, acknowledging the smaller numbers available for the analysis in some of these subgroups. Similar to the total sample, sensory neuropathy symptom clusters, motor-sensory neuropathy symptom clusters, sensorimotor neuropathy symptom clusters, and autonomic neuropathy symptom clusters were also identified in the three subgroups of a) docetaxel, b) paclitaxel or carboplatin plus paclitaxel, and c) cisplatin or carboplatin at most of the assessment time points (Additional file 1: Table S1, Additional file 2: Table S2, Additional file 3: Table S3). Multivariate regression models indicated that race, age, gender, cancer stage, and treatment intent were influence factors for CIPN symptom clusters, with race being the most prominent over time (Additional file 4: Table S4-S7).

## Discussion

To our knowledge, this study is the first to depict the phenotype and trajectories of CIPN through symptom cluster analysis using longitudinal data. The findings illustrated the relationship and development pattern among the diverse symptoms associated with CIPN over time, which have not been determined by previous research. This exploration of the interrelationships of symptoms linked with CIPN has also allowed us to refine and redefine what CIPN is, particularly around the mixed sensorimotor experience and the less common autonomic symptoms. The deeper understanding of CIPN during the course of chemotherapy and one-year follow-up period will help us develop more targeted symptom management strategies to meet the needs of cancer patients.

### Concept of CIPN symptom clusters

This study identified four symptom clusters of CIPN, namely the sensory neuropathy symptom cluster, the motor-sensory neuropathy symptom cluster, the sensorimotor neuropathy symptom cluster, and the autonomic neuropathy symptom cluster, in cancer patients treated with neurotoxic chemotherapy agents from baseline to 12-month follow-up. Sensory neuropathy symptoms were predominant in half of the CIPN symptom clusters. No pure motor but a mixed motor-sensory neuropathy symptom cluster was identified, which may indicate a significant impact of sensory neuropathy symptoms in CIPN. Before the initiation of chemotherapy, the four symptom clusters were also identified and this may be related either to the specific scale used or to pre-existing symptomatology [22].

Sensory nerves are most commonly affected in cancer patients treated with neurotoxic agents and cause various sensory symptoms [3]. These patients often experience tingling and numbness in the hands and/or feet even for a long period [5, 23, 24]. This was confirmed by this study as a stable sensory neuropathy symptom cluster was identified with tingling in the feet, tingling in the hands, and numbness in the feet presented as the core symptoms over time. As a secondary symptom, burning pain in the hands and/or feet only presented at the early two assessment time points in the sensory neuropathy symptom cluster. This may be partially explained by Wolf et al.’s [5] study, which indicated that burning pain in hands and/or feet is less common and does not necessarily exist together with numbness and tingling. However, in our study, the symptoms of burning pain in hands and/or feet were identified in the motor-sensory and the sensorimotor neuropathy symptom clusters rather than completely disappearing. Similarly, the symptoms of cramps in hands and/or feet occurred in the sensory neuropathy symptom cluster at the time points from T1 to T3 but was more prominent in the sensorimotor neuropathy symptom cluster as sensory neuropathy became more stabilized. This may indicate an association between the development of sensory and motor neuropathy symptoms. Future research examining the underlying mechanisms of neuropathy and the associations between CIPN signs and symptoms is warranted.

The motor-sensory neuropathy symptom cluster was defined based on its inclusion of two motor neuropathy symptoms as the core symptoms and several sensory neuropathy symptoms as important secondary symptoms. As was similarly indicated in a previous study, both sensory and motor dysfunctions were detected in cancer patients with established CIPN [25]. However, the relationship between sensory and motor symptoms of CIPN is not clear. As sensory symptoms were predominant and occurred earlier in patients with established CIPN [1, 26], the motor symptoms were possibly a result of prolonged or worsening sensory symptoms of CIPN.

The sensorimotor neuropathy symptom cluster was named as such because it had a sensory neuropathy symptom as its single core symptom but simultaneously contained both motor and sensory neuropathy symptoms as secondary symptoms over time. The secondary symptoms of the sensorimotor neuropathy symptom cluster were flexible and affected both hands and feet; this may reflect an association between the symptoms in hands and feet. However, such association is not high according to Wolf et al.’s [5] report, which demonstrated a difference in patients’ experience of CIPN symptoms between hand and feet.

The last symptom cluster identified by this study was the autonomic neuropathy symptom cluster, with blurred vision serving as the single core symptom. Blurred vision often happens when the retina and optic nerves are affected by neurotoxic agents [27, 28]. Having difficulty hearing was a frequently identified secondary symptom in the autonomic neuropathy symptom cluster. Such an ototoxic effect has been reported in cancer patients treated with platinum and taxanes [29, 30]. It may be caused by damage in the Corti, lateral wall of inner ear, auditory nerve fibers, or spiral ganglion neurons [29]. Other commonly found secondary symptoms included having difficulty in remembering things and having difficulty in concentration. This interesting finding may be linked with chemotherapy-related cognitive impairment or “chemo brain” [31]. Future research is needed to explore the underlying mechanisms of the cognitive symptoms presented in the cluster. However, the autonomic neuropathy symptom cluster was not stable as its internal consistency was moderately low at six out of the ten time points. Furthermore, the core symptom (i.e., blurred vision) had only moderate loadings (< 0.60) at half of the time points. The instability in symptoms, lower item loadings or lower reliability values in this cluster may indicate that autonomic dysfunction as a result of CIPN is not clear or certain, and results perhaps are linked with other treatment-related symptomatology or earlier/pre-existing conditions, not commonly manifested as a result of CIPN. This hypothesis, however, requires further verification.

Subgroup analysis confirmed the presence of these symptom clusters. Each chemotherapy group also manifested its unique feature in symptom clusters. For instance, motor-sensory neuropathy symptom cluster explained higher percentage of variance in the docetaxel subgroup. However, patterns of symptom clusters regarding individual chemotherapy agents were not clearly identified due to insufficient sample size. Future studies focusing on specific chemotherapy agents should be carried out to understand the morphology of CIPN symptom clusters when different chemotherapy protocols are used. Moreover, CIPN symptom clusters may vary between different races. Our data demonstrated that, compared with non-Chinese Asians and Caucasian, Chinese patients experienced less severe CIPN symptom clusters. Nevertheless, the result may be biased due to the high proportion of Chinese patients in the study sample. Prospective study using more balanced sample would help address this question.

### Methodological concerns in the symptom cluster analysis

In terms of methodology, we only used principal component analysis to detect potential symptom clusters of CIPN in cancer patients treated with neurotoxic chemotherapy agents based on the observed variables in our dataset. As this study aimed to provide preliminary information for further research, a confirmatory analysis has not been conducted at present. Some of the symptom clusters identified in this study are not stable, and the structure of symptom clusters at several assessment time points varied largely. These are, to some extent, related to the approach of principal component analysis with varimax rotation used in the study, because it is a dimension reduction technique without assuming any relationship among the symptoms as well as symptom clusters [32, 33]. Currently, the mechanisms of CIPN are not fully discovered [1]. Although certain relationships between CIPN symptoms like numbness, tingling, and shooting/burning pain have been demonstrated in previous studies, information on broader relationships among all the CIPN symptoms are still unclear [5]. Hence, it is unsuitable to presuppose a theoretical model for guiding the present symptom cluster analysis. Future biological research to identify the mechanisms and relationships underlying symptoms and symptom clusters of CIPN is needed.

This study adopted a one-way symptom cluster analysis, and the results were generated from a sequence of cross-sectional analyses. This may not fully reflect longitudinal development of trajectories and the potential mechanisms of each symptom cluster experienced by patients [33]. However, it provides a clearer and broader picture of changes in the component and structure within symptom clusters at each time point than modeling techniques [11]. Better mathematical algorithms and analytical techniques are needed for future symptom cluster research.

Another methodological limitation is that the measurements used in the study were patient-reported outcomes that assess CIPN symptoms. It is worth noting that objective measured outcomes are also important in CIPN. For example, patients with CIPN are likely to have abnormal Achilles tendon reflex, which may be a cause of motor impairment [25]. In addition, objectively measured balance, gait speed, and gait pattern are closely related to physical function and risk of falls in patients with CIPN and therefore should be included in symptom cluster analysis [34]. Results of nerve conduction studies like amplitude and conduction velocity are also significant indicators of axonal damage in CIPN [3]. Therefore, more considerations are needed to determine the use of these objective outcomes in symptom cluster research in CIPN.

### Clinical implications

The existence of relevant symptom clusters indicates the importance of comprehensive and real-time assessment for cancer patients with CIPN, which may enable clinicians to identify major symptoms while fully understanding the dynamic changes of other correlated symptoms over time [10]. In terms of symptom assessment, the use of validated tools with adequate symptom items like the EORTC QLQ-C30 and CIPN20 should be considered in future clinical practice. Given the predominance of sensory neuropathy in CIPN symptom clusters, more emphasis should be placed on evaluating the impact of sensory symptoms on cancer patients with CIPN.

As a total of four symptom clusters of CIPN were identified in this study, it is necessary to organize holistic interventions that targets each symptom cluster simultaneously. An evidence-based care bundle may be promising to manage the multidimensional symptom clusters of CIPN [35]. It is also necessary to adjust intervention plans timely according to the change of symptoms associated with CIPN. Although sensory symptoms are predominant and were widely identified in all of the four CIPN symptom clusters, it is unclear whether managing the sensory neuropathy symptom cluster can simultaneously relieve the motor and autonomic changes. Future research to test this hypothesis is warranted. There are some questions that remain to be answered:

- What are the relationships between sensory and motor neuropathy symptoms?
- What are the relationships between the CIPN symptom clusters?
- What are the relationships between core symptoms and secondary symptoms within a CIPN symptom cluster?
- What are the mechanisms underlying the change of CIPN symptom clusters over time?
- Will change of measurement instruments alter the results of CIPN symptom clusters?
- Which CIPN symptom or symptom cluster should be priority in symptom management?

### Limitations

Certain limitations of this study should be mentioned. First, this study is a secondary analysis, and the sample size for subgroup analysis was not planned in the prior design. Therefore, the findings of symptom clusters in subgroup analysis with individual chemotherapy agents were not reliable at time. These should be addressed in subsequent research. Second, the study included patients with mixed cancer diagnosis, thus leading to a heterogeneous sample, although this may provide a broader picture of CIPN symptom clusters in a wider cancer population. Lastly, patients in the study were treated with different chemotherapy agents and regimen (e.g., weekly versus three weekly) and had completed different number of cycles of chemotherapy, which may influence the patterns of CIPN symptom clusters.

## Conclusions

Results from this study allow us to redefine CIPN. This study identified that CIPN is predominantly a sensory neuropathy either purely sensory or more often mixed sensory-motor neuropathy. There does not seem to be a pure motor neuropathy. Autonomic changes are evident but less clear in this group of patients with cancer. Motor changes in mixed clusters could be either motor neuropathy or, more likely, motor-related changes as a result of prolonged sensory dysfunction. The morphology of CIPN symptom clusters can help us understand the underlying mechanisms and the symptom associations better, and this may enhance CIPN-related symptom management interventions in the future.

## Supplementary information

**Additional file 1:** **Table S1.** Symptom clusters of the docetaxel subgroup over time.

**Additional file 2:** **Table S2.** Symptom clusters of the paclitaxel and carboplatin-paclitaxel subgroups over time.

**Additional file 3:** **Table S3.** Symptom clusters of the cisplatin and carboplatin subgroups over time.

**Additional file 4:** **Table S4.** Influencing factors for the sensory neuropathy symptom cluster over time. **Table S5.** Influencing factors for the motor-sensory neuropathy symptom cluster over time. **Table S6.** Influencing factors for the sensorimotor neuropathy symptom cluster over time. **Table S7.** Influencing factors for the autonomic neuropathy symptom cluster over time.

## Data Availability

The dataset used for the secondary analysis is available from the corresponding author on reasonable request.

## Abbreviations

CIPN: Chemotherapy-induced peripheral neuropathy
EORTC QLQ-C30: European Organization for the Research and Treatment of Cancer Quality of Life Questionnaire-Core 30
EORTC QLQ-CIPN20: European Organization for the Research and Treatment of Cancer Quality of Life Questionnaire-Chemotherapy-Induced Peripheral Neuropathy 20
KMO: Kaiser-Meyer-Olkin measure of sampling adequacy
QoL: Quality-of-life
