# Supplementary material for: Redefining chemotherapy-induced peripheral neuropathy through symptom cluster analysis and patient-reported outcome data over time
Source: BMC Cancer. 2019 Nov 27;19:1151. doi: 10.1186/s12885-019-6352-3 (PMC6882224; doi:10.1186/s12885-019-6352-3)
Supplement: Supplementary file 1 — Additional file 1: Table S1. Symptom clusters of the docetaxel subgroup over time. [file 12885_2019_6352_MOESM1_ESM.docx]

**Table S1** Symptom clusters of docetaxel subgroup over time

| Baseline | KMO = 0.60 | | Cycle1 | KMO = - | | Cycle2 | KMO = 0.64 | | Cycle3 | KMO = 0.71 | | Cycle4 | KMO = 0.67 | | Cycle5 | KMO = 0.48 | | Cycle6 | KMO = 0.32 | |
| --- | --- | --- | --- | --- | --- | --- | --- | --- | --- | --- | --- | --- | --- | --- | --- | --- | --- | --- | --- | --- |
| Items | Factor loadings | Explained (%) | Items | Factor loadings | Explained (%) | Items | Factor loadings | Explained (%) | Items | Factor loadings | Explained (%) | Items | Factor loadings | Explained (%) | Items | Factor loadings | Explained (%) | Items | Factor loadings | Explained (%) |
| **Manipulating small objects** | 0.87 | 9.74 | **Manipulating small objects** | 0.80 | 13.71 | **Manipulating small objects** | 0.63 | 10.68 | **Manipulating small objects** | 0.75 | 11.34 | **Manipulating small objects** | 0.87 | 10.87 | **Manipulating small objects** | 0.84 | 17.54 | **Manipulating small objects** | 0.76 | 24.91 |
| Opening jar | 0.42 |  | Standing/walking feeling ground | 0.93 |  | Opening jar | 0.68 |  | Opening jar | 0.62 |  | Opening jar | 0.54 |  | Holding pen | 0.69 |  | Opening jar | 0.76 |  |
| Holding pen | 0.88 |  | Walking feet drop | 0.93 |  | Burning pain in hands | 0.89 |  | Holding pen | 0.83 |  | Holding pen | 0.84 |  | Irritable | 0.86 |  | Holding pen | 0.89 |  |
|  | α = 0.40 |  | Numbness in feet | 0.65 |  | Tingling hands | 0.82 |  | Numbness in hands | 0.80 |  | Distinguishing hot-cold | 0.77 |  | Depressed | 0.85 |  | Irritable | 0.88 |  |
|  |  |  | Tingling feet | 0.59 |  | Burning pain in feet | 0.59 |  | Numbness in feet | 0.64 |  | Cramps in hands | 0.75 |  | Worry | 0.85 |  | Worry | 0.85 |  |
|  |  |  | Climbing stairs | 0.50 |  | Tingling feet | 0.48 |  | Tingling hands | 0.58 |  | Burning pain in hands | 0.69 |  | Walking feet drop | 0.75 |  | Walking feet drop | 0.84 |  |
|  |  |  |  | α = 0.79 |  |  | α = 0.82 |  | Distinguishing hot-cold | 0.53 |  |  | α = 0.82 |  | Tense | 0.74 |  | Depressed | 0.83 |  |
|  |  |  |  |  |  |  |  |  |  | α = 0.82 |  |  |  |  | Difficulty remembering | 0.49 |  | Tense | 0.80 |  |
|  |  |  |  |  |  |  |  |  |  |  |  |  |  |  |  | α = 0.92 |  | Climbing stairs | 0.65 |  |
|  |  |  |  |  |  |  |  |  |  |  |  |  |  |  |  |  |  | Blurred vision | 0.62 |  |
|  |  |  |  |  |  |  |  |  |  |  |  |  |  |  |  |  |  | Numbness in hands | 0.61 |  |
|  |  |  |  |  |  |  |  |  |  |  |  |  |  |  |  |  |  | Standing/walking feeling ground | 0.59 |  |
|  |  |  |  |  |  |  |  |  |  |  |  |  |  |  |  |  |  | Dizzy | 0.59 |  |
|  |  |  |  |  |  |  |  |  |  |  |  |  |  |  |  |  |  | Appetite loss | 0.52 |  |
|  |  |  |  |  |  |  |  |  |  |  |  |  |  |  |  |  |  | Difficulty remembering | 0.48 |  |
|  |  |  |  |  |  |  |  |  |  |  |  |  |  |  |  |  |  |  | α = 0.94 |  |
|  |  |  |  |  |  |  |  |  |  |  |  |  |  |  |  |  |  |  |  |  |
| **Walking feet drop** | **0.89** | 7.70 | - |  |  | **Walking feet drop** | **0.72** | 6.58 | **Walking feet drop** | **0.84** | 7.59 | **Walking feet drop** | **0.72** | 6.79 | **Standing/walking feeling ground** | **0.58** | 7.68 | **-** |  |  |
| Burning pain in feet | 0.76 |  |  |  |  | Cramps in feet | .856 |  | **Standing/walking feeling ground** | **0.78** |  | **Standing/walking feeling ground** | **0.71** |  | Cramps in hands | 0.78 |  |  |  |  |
| Tingling feet | 0.73 |  |  |  |  |  | α = 0.79 |  | Burning pain in feet | 0.70 |  |  | α = 0.82 |  | Cramps in feet | 0.61 |  |  |  |  |
|  | α = 0.75 |  |  |  |  |  |  |  |  | α = 0.85 |  |  |  |  | Numbness in feet | 0.52 |  |  |  |  |
|  |  |  |  |  |  |  |  |  |  |  |  |  |  |  |  | α = 0.79 |  |  |  |  |
|  |  |  |  |  |  |  |  |  |  |  |  |  |  |  |  |  |  |  |  |  |
| **Dizzy** | **0.72** | 7.33 | **Dizzy** | **0.57** | **5.80** | **Dizzy** | **.604** | **6.66** | **Dizzy** | **0.54** | **6.43** | **Dizzy** | **0.67** | **7.21** | **Dizzy** | **0.69** | **12.32** | **-** |  |  |
| **Blurred vision** | **0.99** |  | **Blurred vision** | **0.36** |  | Standing/walking feeling ground | .827 |  | Nausea | 0.86 |  | **Blurred vision** | **0.47** |  | **Blurred vision** | **0.55** |  |  |  |  |
| Climbing stairs | 0.99 |  | Difficulty remembering | 0.73 |  | Climbing stairs | .581 |  | Appetite loss | 0.65 |  | Burning pain in feet | 0.78 |  | Trouble sleeping | 0.83 |  |  |  |  |
|  | α = 0.90 |  | Difficulty concentration | 0.66 |  |  | α = 0.68 |  | Trouble sleeping | 0.36 |  | Difficulty hearing | 0.63 |  | Rest | 0.77 |  |  |  |  |
|  |  |  |  | α = 0.63 |  |  |  |  |  | α = 0.61 |  |  | α = 0.63 |  | Weak | 0.64 |  |  |  |  |
|  |  |  |  |  |  |  |  |  |  |  |  |  |  |  | Opening jar | 0.63 |  |  |  |  |
|  |  |  |  |  |  |  |  |  |  |  |  |  |  |  | Difficulty concentration | 0.56 |  |  |  |  |
|  |  |  |  |  |  |  |  |  |  |  |  |  |  |  | Pain | 0.46 |  |  |  |  |
|  |  |  |  |  |  |  |  |  |  |  |  |  |  |  |  | α = 0.86 |  |  |  |  |
|  |  |  |  |  |  |  |  |  |  |  |  |  |  |  |  |  |  |  |  |  |
| **Numbness in hands** | **0.80** | 6.94 | **Numbness in hands** | **0.62** | 8.37 | **Numbness in hands** | **0.54** | 5.58 | Cramps in hands | 0.84 | 7.76 | **Numbness in hands** | **0.49** | 7.38 | **Numbness in hands** | **0.68** | 5.92 | **Burning pain in feet** | **0.88** | 16.86 |
| Cramps in hands | 0.69 |  | Cramps in hands | 0.75 |  | Distinguishing hot-cold | 0.72 |  | Burning pain in hands | 0.83 |  | Tingling feet | 0.84 |  | Distinguishing hot-cold | 0.69 |  | Diarrhea | 0.83 |  |
| Tingling hands | 0.63 |  | Burning pain in feet | 0.75 |  | Numbness in feet | 0.49 |  | Cramps in feet | 0.64 |  | Numbness in feet | 0.77 |  |  | α = 0.46 |  | Burning pain in hands | 0.82 |  |
| Burning pain in hands | 0.58 |  | Tingling hands | 0.61 |  | Difficulty concentration | 0.43 |  |  | α = 0.76 |  | Tingling hands | 0.65 |  |  |  |  | Tingling hands | 0.78 |  |
|  | α = 0.72 |  | Burning pain in hands | 0.55 |  |  | α = 0.44 |  |  |  |  |  | α = 0.76 |  |  |  |  | Constipation | 0.73 |  |
|  |  |  |  | α = 0.78 |  |  |  |  |  |  |  |  |  |  |  |  |  | Tingling feet | 0.73 |  |
|  |  |  |  |  |  |  |  |  |  |  |  |  |  |  |  |  |  | Nausea | 0.70 |  |
|  |  |  |  |  |  |  |  |  |  |  |  |  |  |  |  |  |  | Numbness in feet | 0.68 |  |
|  |  |  |  |  |  |  |  |  |  |  |  |  |  |  |  |  |  |  | α = 0.90 |  |
|  |  |  |  |  |  |  |  |  |  |  |  |  |  |  |  |  |  |  |  |  |
| Cramps in feet | 0.73 | 4.44 | Cramps in feet | 0.64 | 6.24 |  |  |  | Climbing stairs | 0.73 | 9.79 | Climbing stairs | 0.69 | 9.13 | Climbing stairs | 0.62 | 7.75 | Distinguishing hot-cold | 0.83 | 6.54 |
| Numbness in feet | 0.58 |  | Opening jar | 0.40 |  |  |  |  | Short of breath | 0.72 |  | Rest | 0.77 |  | Short of breath | 0.76 |  | Vomiting | 0.60 |  |
|  | α = 0.35 |  | Vomiting | 0.76 |  |  |  |  | Tired | 0.69 |  | Weak | 0.65 |  | Tired | 0.64 |  |  | α = 0.64 |  |
|  |  |  | Nausea | 0.68 |  |  |  |  | Weak | 0.62 |  | Tired | 0.63 |  |  | α = 0.77 |  |  |  |  |
|  |  |  |  | α = 0.62 |  |  |  |  | Rest | 0.58 |  | Trouble sleeping | 0.61 |  |  |  |  |  |  |  |
|  |  |  |  |  |  |  |  |  | Pain | 0.52 |  | Short of breath | 0.61 |  |  |  |  |  |  |  |
|  |  |  |  |  |  |  |  |  |  | α = 0.82 |  |  | α = 0.81 |  |  |  |  |  |  |  |
|  |  |  |  |  |  |  |  |  |  |  |  |  |  |  |  |  |  |  |  |  |
| Difficulty hearing | 0.88 | 4.13 | Difficulty hearing | .802 | 3.93 | Difficulty hearing | 0.59 | 5.79 | Difficulty hearing | 0.81 | 6.69 | Vomiting | 0.83 | 6.97 | Difficulty hearing | -0.63 | 4.26 |  |  |  |
| Diarrhea | 0.73 |  | Appetite loss | .514 |  | Short of breath | 0.74 |  | Tingling feet | 0.64 |  | Nausea | 0.69 |  | Constipation | 0.73 |  |  |  |  |
|  | α = 0.01 |  |  | α = 0.00 |  |  | α = 0.43 |  | Blurred vision | 0.52 |  | Appetite loss | 0.64 |  |  | α = -0.40 |  |  |  |  |
|  |  |  |  |  |  |  |  |  |  | α = 0.60 |  | Cramps in feet | 0.40 |  |  |  |  |  |  |  |
|  |  |  |  |  |  |  |  |  |  |  |  |  | α = 0.60 |  |  |  |  |  |  |  |
|  |  |  |  |  |  |  |  |  |  |  |  |  |  |  |  |  |  |  |  |  |
| Distinguishing hot-cold | 0.90 |  |  |  |  | Holding pen | 0.75 | 4.10 |  |  |  |  |  |  | Burning pain in hands | 0.88 | 11.56 | Cramps in hands | 0.91 | 5.33 |
| Tired | 0.38 |  |  |  |  | Trouble sleeping | -0.56 |  |  |  |  |  |  |  | Burning pain in feet | 0.79 |  | Cramps in feet | 0.89 |  |
|  | α = 0.09 |  |  |  |  |  | α = -0.13 |  |  |  |  |  |  |  | Diarrhea | 0.78 |  |  | α = 0.80 |  |
|  |  |  |  |  |  |  |  |  |  |  |  |  |  |  | Vomiting | 0.75 |  |  |  |  |
|  |  |  |  |  |  |  |  |  |  |  |  |  |  |  |  | α = 0.89 |  |  |  |  |
|  |  |  |  |  |  |  |  |  |  |  |  |  |  |  |  |  |  |  |  |  |
|  |  |  |  |  |  |  |  |  |  |  |  |  |  |  | Tingling feet | 0.84 | 5.97 |  |  |  |
|  |  |  |  |  |  |  |  |  |  |  |  |  |  |  | Tingling hands | 0.70 |  |  |  |  |
|  |  |  |  |  |  |  |  |  |  |  |  |  |  |  |  | α = 0.72 |  |  |  |  |
|  |  |  |  |  |  |  |  |  |  |  |  |  |  |  |  |  |  |  |  |  |

| 6MFU | KMO = 0.76 | | 9MFU | KMO = 0.64 | | 12MFU | KMO = 0. 66 | |
| --- | --- | --- | --- | --- | --- | --- | --- | --- |
| Items | Factor loadings | Explained (%) | Items | Factor loadings | Explained (%) | Items | Factor loadings | Explained (%) |
| **Manipulating small objects** | **0.84** | **16.46** | **Manipulating small objects** | **0.82** | **8.94** | **Manipulating small objects** | **0.82** | **7.31** |
| Opening jar | 0.75 |  | Opening jar | 0.76 |  | Opening jar | 0.66 |  |
| Holding pen | 0.81 |  | Holding pen | 0.86 |  | Difficulty remembering | 0.57 |  |
| Depressed | 0.83 |  | Burning pain in hands | 0.48 |  |  | α = 0.55 |  |
| Worry | 0.75 |  |  | α = 0.81 |  |  |  |  |
| Irritable | 0.73 |  |  |  |  |  |  |  |
| Tense | 0.71 |  |  |  |  |  |  |  |
| Distinguishing hot-cold | 0.55 |  |  |  |  |  |  |  |
| Pain | 0.45 |  |  |  |  |  |  |  |
|  | α = 0.91 |  |  |  |  |  |  |  |
|  |  |  |  |  |  |  |  |  |
|  |  |  |  |  |  |  |  |  |
|  |  |  |  |  |  |  |  |  |
| **Walking feet drop** | **0.86** | **8.47** | **Walking feet drop** | **0.85** | **13.58** | **Walking feet drop** | **0.91** | 8.77 |
| **Standing/walking feeling ground** | **0.81** |  | **Standing/walking feeling ground** | **0.84** |  | **Standing/walking feeling ground** | **0.85** |  |
| Climbing stairs | 0.74 |  | Burning pain in feet | 0.82 |  | Climbing stairs | 0.73 |  |
|  | α = 0.94 |  | Climbing stairs | 0.77 |  | Numbness in feet | 0.44 |  |
|  |  |  | Pain | 0.57 |  |  | α = 0.85 |  |
|  |  |  | Appetite loss | 0.56 |  |  |  |  |
|  |  |  | Short of breath | 0.49 |  |  |  |  |
|  |  |  |  | α = 0.85 |  |  |  |  |
|  |  |  |  |  |  |  |  |  |
| **Dizzy** | **0.73** | **8.97** | **Dizzy** | **0.60** | **5.48** | **Dizzy** | **0.99** | **5.96** |
| **Blurred vision** | **0.69** |  | **Blurred vision** | **0.51** |  | **Blurred vision** | **0.99** |  |
| Difficulty hearing | 0.76 |  | Difficulty hearing | 0.78 |  |  | α = 1.00 |  |
| Difficulty remembering | 0.50 |  |  | α = 0.71 |  |  |  |  |
| Weak | 0.48 |  |  |  |  |  |  |  |
| Short of breath | 0.44 |  |  |  |  |  |  |  |
|  | α = 0.74 |  |  |  |  |  |  |  |
|  |  |  |  |  |  |  |  |  |
| **Numbness in hands** | **0.60** | 11.99 | **Numbness in hands** | **-0.52** | 4.22 | **Numbness in hands** | **0.49** | 18.49 |
| Tingling hands | 0.81 |  | Numbness in feet | 0.75 |  | Nausea | 0.90 |  |
| Tingling feet | 0.80 |  |  | α = -0.00 |  | Tingling hands | 0.89 |  |
| Numbness in feet | 0.76 |  |  |  |  | Tingling feet | 0.87 |  |
| Burning pain in feet | 0.75 |  |  |  |  | Vomiting | 0.84 |  |
| Burning pain in hands | 0.66 |  |  |  |  | Appetite loss | 0.83 |  |
| Diarrhea | 0.63 |  |  |  |  | Cramps in feet | 0.75 |  |
|  | α = 0.85 |  |  |  |  | Weak | 0.62 |  |
|  |  |  |  |  |  | Holding pen | 0.54 |  |
|  |  |  |  |  |  | Short of breath | 0.47 |  |
|  |  |  |  |  |  |  | α = 0.90 |  |
|  |  |  |  |  |  |  |  |  |
| Cramps in hands | 0.79 | 8.20 | Cramps in hands | 0.68 | 7.80 | Cramps in hands | 0.77 | 4.55 |
| Nausea | 0.71 |  | Tingling hands | 0.88 |  | Burning pain in hands | 0.81 |  |
| Vomiting | 0.65 |  | Tingling feet | 0.60 |  |  | α = 0.39 |  |
| Appetite loss | 0.64 |  | Nausea | 0.50 |  |  |  |  |
|  | α = 0.68 |  |  | α = 0.70 |  |  |  |  |
|  |  |  |  |  |  |  |  |  |
|  |  |  | Diarrhea | 0.95 | 10.42 | Depressed | 0.80 | 8.04 |
|  |  |  | Vomiting | 0.91 |  | Irritable | 0.71 |  |
|  |  |  | Constipation | 0.78 |  | Burning pain in feet | 0.64 |  |
|  |  |  | Distinguishing hot-cold | 0.62 |  | Tired | 0.49 |  |
|  |  |  |  | α = 0.85 |  | Difficulty concentration | 0.47 |  |
|  |  |  |  |  |  |  | α = 0.74 |  |
|  |  |  |  |  |  |  |  |  |
|  |  |  |  |  |  |  |  |  |
|  |  |  |  |  |  |  |  |  |
|  |  |  |  |  |  |  |  |  |
