# Supplementary material for: Redefining chemotherapy-induced peripheral neuropathy through symptom cluster analysis and patient-reported outcome data over time
Source: BMC Cancer. 2019 Nov 27;19:1151. doi: 10.1186/s12885-019-6352-3 (PMC6882224; doi:10.1186/s12885-019-6352-3)
Supplement: Supplementary file 2 — Additional file 2: Table S2. Symptom clusters of the paclitaxel and carboplatin-paclitaxel subgroups over time. [file 12885_2019_6352_MOESM2_ESM.docx]

**Table S2** Symptom clusters of paclitaxel and carboplatin-paclitaxel subgroups over time

| Baseline | KMO = - | | Cycle1 | KMO = 0.61 | | Cycle2 | KMO = 0.75 | | Cycle3 | KMO = 0.78 | | Cycle4 | KMO = 0.74 | | Cycle5 | KMO = 0.41 | | Cycle6 | KMO = 0.40 | |
| --- | --- | --- | --- | --- | --- | --- | --- | --- | --- | --- | --- | --- | --- | --- | --- | --- | --- | --- | --- | --- |
| Items | Factor loadings | Explained (%) | Items | Factor loadings | Explained (%) | Items | Factor loadings | Explained (%) | Items | Factor loadings | Explained (%) | Items | Factor loadings | Explained (%) | Items | Factor loadings | Explained (%) | Items | Factor loadings | Explained (%) |
| **-** |  |  | **Tingling feet** | 0.91 | 7.27 | **Tingling feet** | 0.89 | 11.48 | **Tingling feet** | 0.88 | 11.77 | **Tingling feet** | 0.84 | 11.48 | **Tingling feet** | 0.82 | 7.16 | **Tingling feet** | 0.87 | 10.04 |
|  |  |  | **Tingling hands** | 0.85 |  | **Tingling hands** | 0.84 |  | **Tingling hands** | 0.81 |  | **Tingling hands** | 0.80 |  | **Tingling hands** | 0.88 |  | **Tingling hands** | 0.91 |  |
|  |  |  | **Numbness in feet** | 0.57 |  | **Numbness in feet** | 0.82 |  | **Numbness in feet** | 0.78 |  | **Numbness in feet** | 0.75 |  |  | α = 0.85 |  | Numbness in feet | 0.59 |  |
|  |  |  |  | α = 0.78 |  | **Numbness in hands** | 0.80 |  | **Numbness in hands** | 0.73 |  | **Numbness in hands** | 0.81 |  |  |  |  | Numbness in hands | 0.69 |  |
|  |  |  |  |  |  | Cramps in feet | 0.48 |  | Standing/walking feeling ground | 0.44 |  | Burning pain in hands | 0.60 |  |  |  |  |  | α = 0.89 |  |
|  |  |  |  |  |  |  | α = 0.88 |  |  | α = 0.89 |  | Burning pain in feet | 0.60 |  |  |  |  |  |  |  |
|  |  |  |  |  |  |  |  |  |  |  |  |  | α = 0.88 |  |  |  |  |  |  |  |
|  |  |  |  |  |  |  |  |  |  |  |  |  |  |  |  |  |  |  |  |  |
|  |  |  | **Manipulating small objects** | 0.66 | 4.81 | **Manipulating small objects** | 0.77 | 6.91 | **Manipulating small objects** | 0.85 | 8.55 | **Manipulating small objects** | 0.79 | 6.58 | **Manipulating small objects** | 0.92 | 14.75 | **Manipulating small objects** | 0.78 | 15.41 |
|  |  |  | Opening jar | 0.43 |  | **Holding pen** | 0.76 |  | **Holding pen** | 0.85 |  | **Holding pen** | 0.82 |  | **Holding pen** | 0.90 |  | **Holding pen** | 0.74 |  |
|  |  |  | Constipation | -0.50 |  | Distinguishing hot-cold | 0.59 |  | Distinguishing hot-cold | 0.72 |  |  | α = 0.90 |  | Distinguishing hot-cold | 0.88 |  | Opening jar | 0.75 |  |
|  |  |  |  | α = 0.17 |  | Difficulty hearing | 0.55 |  |  | α = 0.84 |  |  |  |  | Worry | 0.64 |  | Difficulty hearing | 0.82 |  |
|  |  |  |  |  |  |  | α = 0.69 |  |  |  |  |  |  |  | Tense | 0.62 |  | Burning pain in hands | 0.81 |  |
|  |  |  |  |  |  |  |  |  |  |  |  |  |  |  | Difficulty concentration | 0.60 |  | Distinguishing hot-cold | 0.78 |  |
|  |  |  |  |  |  |  |  |  |  |  |  |  |  |  | Difficulty remembering | 0.46 |  |  | α = 0.91 |  |
|  |  |  |  |  |  |  |  |  |  |  |  |  |  |  |  | α = 0.90 |  |  |  |  |
|  |  |  |  |  |  |  |  |  |  |  |  |  |  |  |  |  |  |  |  |  |
|  |  |  | **Blurred vision** | 0.56 | 11.50 | **Blurred vision** | 0.60 | 9.80 | **Blurred vision** | 0.39 | 10.10 | **-** |  |  | **Blurred vision** | 0.65 | 5.41 | **Blurred vision** | 0.63 | 10.12 |
|  |  |  | Dizzy | 0.59 |  | Standing/walking feeling ground | 0.69 |  | Tense | 0.73 |  |  |  |  | Burning pain in hands | 0.83 |  | Difficulty remembering | 0.76 |  |
|  |  |  | Climbing stairs | 0.80 |  | Burning pain in hands | 0.66 |  | Worry | 0.72 |  |  |  |  |  | α = 0.55 |  | Burning pain in feet | 0.75 |  |
|  |  |  | Walking feet drop | 0.72 |  | Burning pain in feet | 0.54 |  | Depressed | 0.67 |  |  |  |  |  |  |  |  | α = 0.77 |  |
|  |  |  | Tired | 0.70 |  |  | α = 0.87 |  | Difficulty concentration | 0.60 |  |  |  |  |  |  |  |  |  |  |
|  |  |  | Burning pain in feet | 0.60 |  |  |  |  | Difficulty remembering | 0.54 |  |  |  |  |  |  |  |  |  |  |
|  |  |  | Standing/walking feeling ground | 0.56 |  |  |  |  |  | α = 0.87 |  |  |  |  |  |  |  |  |  |  |
|  |  |  |  | α = 0.03 |  |  |  |  |  |  |  |  |  |  |  |  |  |  |  |  |
|  |  |  |  |  |  |  |  |  |  |  |  |  |  |  |  |  |  |  |  |  |
|  |  |  | Difficulty remembering | 0.74 | 6.22 | **Climbing stairs** | 0.58 | 10.50 | **Climbing stairs** | 0.47 | 13.65 | **Climbing stairs** | 0.71 | 8.50 | **Climbing stairs** | 0.74 | 11.81 | **Climbing stairs** | 0.53 | 9.56 |
|  |  |  | Holding pen | 0.59 |  | Rest | 0.82 |  | Tired | 0.73 |  | **Standing/walking feeling ground** | 0.71 |  | **Standing/walking feeling ground** | 0.72 |  | **Standing/walking feeling ground** | 0.61 |  |
|  |  |  | Burning pain in hands | 0.55 |  | Tired | 0.77 |  | Rest | 0.73 |  | Walking feet drop | 0.90 |  | Numbness in feet | 0.73 |  | Constipation | 0.67 |  |
|  |  |  |  | α = 0.58 |  | Short of breath | 0.59 |  | Short of breath | 0.72 |  |  | α = 0.85 |  | Burning pain in feet | 0.72 |  | Pain | 0.69 |  |
|  |  |  |  |  |  | Weak | 0.57 |  | Weak | 0.70 |  |  |  |  | Numbness in hands | 0.67 |  |  | α = 0.72 |  |
|  |  |  |  |  |  | Trouble sleeping | 0.55 |  | Pain | 0.66 |  |  |  |  | Tired | 0.62 |  |  |  |  |
|  |  |  |  |  |  |  | α = 0.90 |  | Trouble sleeping | 0.59 |  |  |  |  |  | α = 0.85 |  |  |  |  |
|  |  |  |  |  |  |  |  |  | Dizzy | 0.53 |  |  |  |  |  |  |  |  |  |  |
|  |  |  |  |  |  |  |  |  |  | α = 0.90 |  |  |  |  |  |  |  |  |  |  |
|  |  |  |  |  |  |  |  |  |  |  |  |  |  |  |  |  |  |  |  |  |
|  |  |  | Cramps in feet | 0.82 | 5.71 | Worry | 0.84 | 13.80 | Cramps in hands | 0.79 | 7.32 | Rest | 0.77 | 16.27 | Depressed | 0.85 | 8.97 | Cramps in hands | 0.77 | 7.85 |
|  |  |  | Cramps in hands | 0.80 |  | Irritable | 0.83 |  | Diarrhea | 0.67 |  | Tired | 0.74 |  | Dizzy | 0.77 |  | Walking feet drop | 0.64 |  |
|  |  |  |  | α = 0.69 |  | Depressed | 0.83 |  | Burning pain in hands | 0.56 |  | Weak | 0.68 |  | Opening jar | 0.64 |  | Irritable | 0.42 |  |
|  |  |  |  |  |  | Tense | 0.83 |  |  | α = 0.63 |  | Trouble sleeping | 0.66 |  | Short of breath | 0.50 |  |  | α = 0.65 |  |
|  |  |  |  |  |  | Difficulty remembering | 0.58 |  |  |  |  | Pain | 0.64 |  | Irritable | 0.47 |  |  |  |  |
|  |  |  |  |  |  | Dizzy | 0.44 |  |  |  |  | Dizzy | 0.59 |  |  | α = 0.84 |  |  |  |  |
|  |  |  |  |  |  | Pain | 0.44 |  |  |  |  | Short of breath | 0.58 |  |  |  |  |  |  |  |
|  |  |  |  |  |  |  | α = 0.90 |  |  |  |  | Irritable | 0.56 |  |  |  |  |  |  |  |
|  |  |  |  |  |  |  |  |  |  |  |  | Worry | 0.55 |  |  |  |  |  |  |  |
|  |  |  |  |  |  |  |  |  |  |  |  |  | α = 0.91 |  |  |  |  |  |  |  |
|  |  |  |  |  |  |  |  |  |  |  |  |  |  |  |  |  |  |  |  |  |
|  |  |  | Distinguishing hot-cold | 0.85 | 5.22 | Opening jar | 0.72 | 5.97 | Walking feet drop | 0.72 | 7.08 | Cramps in hands | 0.81 | 15.87 | Cramps in hands | 0.85 | 8.03 | Diarrhea | 0.88 | 5.54 |
|  |  |  | Pain | 0.42 |  | Walking feet drop | 0.53 |  | Difficulty hearing | 0.62 |  | Difficulty hearing | 0.74 |  | Difficulty hearing | 0.71 |  | Dizzy | 0.60 |  |
|  |  |  |  | α = 0.27 |  |  | α = 0.69 |  | Opening jar | 0.60 |  | Nausea | 0.73 |  | Cramps in feet | 0.70 |  |  | α = 0.51 |  |
|  |  |  |  |  |  |  |  |  |  | α = 0.80 |  | Constipation | 0.69 |  |  | α = 0.80 |  |  |  |  |
|  |  |  |  |  |  |  |  |  |  |  |  | Appetite loss | 0.68 |  |  |  |  |  |  |  |
|  |  |  |  |  |  |  |  |  |  |  |  | Cramps in feet | 0.64 |  |  |  |  |  |  |  |
|  |  |  |  |  |  |  |  |  |  |  |  | Depressed | 0.62 |  |  |  |  |  |  |  |
|  |  |  |  |  |  |  |  |  |  |  |  | Tense | 0.60 |  |  |  |  |  |  |  |
|  |  |  |  |  |  |  |  |  |  |  |  |  | α = 0.91 |  |  |  |  |  |  |  |
|  |  |  |  |  |  |  |  |  |  |  |  |  |  |  |  |  |  |  |  |  |
|  |  |  | Short of breath | 0.53 | 5.09 |  |  |  | Vomiting | 0.87 | 10.99 | Difficulty remembering | 0.82 | 6.49 | Appetite loss | 0.75 | 7.42 |  |  |  |
|  |  |  | Diarrhea | 0.49 |  |  |  |  | Nausea | 0.80 |  | Difficulty concentration | 0.62 |  | Walking feet drop | 0.73 |  |  |  |  |
|  |  |  | Numbness in hands | -0.44 |  |  |  |  | Appetite loss | 0.65 |  | Distinguishing hot-cold | 0.46 |  | Diarrhea | 0.69 |  |  |  |  |
|  |  |  | Rest | 0.44 |  |  |  |  | Irritable | 0.56 |  |  | α = 0.72 |  |  | α = 0.74 |  |  |  |  |
|  |  |  |  | α = -0.01 |  |  |  |  | Burning pain in feet | 0.50 |  |  |  |  |  |  |  |  |  |  |
|  |  |  |  |  |  |  |  |  |  | α = 0.86 |  |  |  |  |  |  |  |  |  |  |
|  |  |  |  |  |  |  |  |  |  |  |  |  |  |  |  |  |  |  |  |  |
|  |  |  |  |  |  |  |  |  | Constipation | 0.71 | 5.70 |  |  |  |  |  |  |  |  |  |
|  |  |  |  |  |  |  |  |  | Cramps in feet | 0.65 |  |  |  |  |  |  |  |  |  |  |
|  |  |  |  |  |  |  |  |  |  | α = 0.56 |  |  |  |  |  |  |  |  |  |  |

| 6MFU | KMO = 0.75 | | 9MFU | KMO = 0.71 | | 12MFU | KMO = - | |
| --- | --- | --- | --- | --- | --- | --- | --- | --- |
| Items | Factor loadings | Explained (%) | Items | Factor loadings | Explained (%) | Items | Factor loadings | Explained (%) |
| **Tingling feet** | 0.75 | 14.49 | **Tingling feet** | 0.84 | 11.21 | **Tingling feet** | 0.77 | 6.43 |
| **Tingling hands** | 0.76 |  | **Tingling hands** | 0.86 |  | **Numbness in feet** | 0.75 |  |
| **Numbness in feet** | 0.79 |  | **Numbness in feet** | 0.79 |  |  | α = 0.81 |  |
| **Numbness in hands** | 0.86 |  | **Numbness in hands** | 0.82 |  | **Tingling hands** | 0.69 | 5.41 |
| Manipulating small objects | 0.71 |  |  | α = 0.92 |  | Burning pain in hands | 0.85 |  |
| Cramps in hands | 0.55 |  |  |  |  |  | α = 0.58 |  |
| Cramps in feet | 0.55 |  |  |  |  |  |  |  |
| Holding pen | 0.54 |  |  |  |  |  |  |  |
|  | α = 0.90 |  |  |  |  |  |  |  |
|  |  |  |  |  |  |  |  |  |
| **-** |  |  | **Manipulating small objects** | 0.76 | 7.33 | **Manipulating small objects** | 0.88 | 8.90 |
|  |  |  | **Holding pen** | 0.68 |  | **Holding pen** | 0.78 |  |
|  |  |  | Pain | 0.50 |  | Numbness in hands | 0.73 |  |
|  |  |  |  | α = 0.74 |  |  | α = 0.82 |  |
|  |  |  |  |  |  |  |  |  |
| **Blurred vision** | 0.43 | 8.86 | **Blurred vision** | 0.58 | 10.11 | **Blurred vision** | 0.59 | 4.86 |
| Standing/walking feeling ground | 0.80 |  | Difficulty hearing | 0.82 |  | Difficulty remembering | 0.86 |  |
| Walking feet drop | 0.79 |  | Walking feet drop | 0.76 |  |  | α = 0.46 |  |
| Climbing stairs | 0.66 |  | Climbing stairs | 0.72 |  |  |  |  |
|  | α = 0.80 |  | Standing/walking feeling ground | 0.58 |  |  |  |  |
|  |  |  |  | α = 0.88 |  |  |  |  |
|  |  |  |  |  |  |  |  |  |
| Burning pain in hands | 0.90 | 8.09 | Burning pain in hands | 0.93 | 8.53 | **Climbing stairs** | 0.80 | 15.31 |
| Burning pain in feet | 0.83 |  | Burning pain in feet | 0.91 |  | **Standing/walking feeling ground** | 0.90 |  |
| Opening jar | 0.54 |  | Opening jar | 0.76 |  | Walking feet drop | 0.86 |  |
| Pain | 0.42 |  |  | α = 0.87 |  | Dizzy | 0.83 |  |
|  | α = 0.76 |  |  |  |  | Opening jar | 0.68 |  |
|  |  |  |  |  |  | Cramps in feet | 0.65 |  |
|  |  |  |  |  |  |  | α = 0.92 |  |
|  |  |  |  |  |  |  |  |  |
| Dizzy | 0.64 | 6.23 | Worry | 0.86 | 14.91 | Burning pain in feet | 0.68 | 4.97 |
| Appetite loss | 0.64 |  | Irritable | 0.83 |  | Cramps in hands | 0.59 |  |
| Nausea | 0.53 |  | Depressed | 0.80 |  |  | α = 0.64 |  |
|  | α = 0.81 |  | Tense | 0.79 |  |  |  |  |
|  |  |  | Cramps in feet | 0.51 |  |  |  |  |
|  |  |  | Tired | 0.51 |  |  |  |  |
|  |  |  | Difficulty remembering | 0.46 |  |  |  |  |
|  |  |  | Cramps in hands | 0.43 |  |  |  |  |
|  |  |  | Constipation | 0.36 |  |  |  |  |
|  |  |  |  | α = 0.90 |  |  |  |  |
|  |  |  |  |  |  |  |  |  |
| Distinguishing hot-cold | 0.75 | 5.24 | Distinguishing hot-cold | 0.88 | 6.02 |  |  |  |
| Difficulty hearing | 0.37 |  | Diarrhea | 0.59 |  |  |  |  |
|  | α = 0.52 |  | Short of breath | 0.54 |  |  |  |  |
|  |  |  |  | α = 0.58 |  |  |  |  |
