# Supplementary material for: Redefining chemotherapy-induced peripheral neuropathy through symptom cluster analysis and patient-reported outcome data over time
Source: BMC Cancer. 2019 Nov 27;19:1151. doi: 10.1186/s12885-019-6352-3 (PMC6882224; doi:10.1186/s12885-019-6352-3)
Supplement: Supplementary file 3 — Additional file 3: Table S3. Symptom clusters of the cisplatin and carboplatin subgroups over time. [file 12885_2019_6352_MOESM3_ESM.docx]

**Table S3** Symptom clusters of cisplatin and carboplatin subgroups over time

| Baseline | KMO: - | | Cycle2 | KMO: - | | 6MFU | KMO: - | |
| --- | --- | --- | --- | --- | --- | --- | --- | --- |
| Items | Factor loadings | Explained (%) | Items | Factor loadings | Explained (%) | Items | Factor loadings | Explained (%) |
| **Tingling feet** | 0.90 | 3.40 | **Tingling feet** | 0.74 | 11.45 | **Tingling feet** | 0.85 | 12.17 |
| **Tingling hands** | 0.87 |  | **Tingling hands** | 0.89 |  | **Tingling hands** | 0.93 |  |
| Burning pain in feet | 0.93 |  | Numbness in feet | 0.85 |  | Numbness in feet | 0.83 |  |
| Burning pain in hands | 0.92 |  | Numbness in hands | 0.83 |  | Numbness in hands | 0.70 |  |
| Blurred vision | 0.67 |  |  | α = 0.88 |  | Distinguishing hot-cold | 0.70 |  |
|  | α = 0.92 |  |  |  |  |  | α = 0.89 |  |
|  |  |  |  |  |  |  |  |  |
| **Manipulating small objects** | 0.76 | 2.91 | **Manipulating small objects** | 0.85 | 6.33 | **Manipulating small objects** | 0.59 | 9.69 |
| Holding pen | 0.64 |  | Opening jar | 0.79 |  | Difficulty concentration | 0.81 |  |
| Cramps in hands | 0.82 |  |  | α = 0.63 |  | Pain | 0.63 |  |
| Dizzy | 0.67 |  |  |  |  | Burning pain in feet | 0.58 |  |
| Numbness in feet | 0.54 |  |  |  |  |  | α = 0.77 |  |
|  | α = 0.83 |  |  |  |  |  |  |  |
|  |  |  |  |  |  |  |  |  |
| - |  |  | **Blurred vision** | 0.54 | 16.52 | **Blurred vision** | 0.71 | 5.80 |
|  |  |  | Worry | 0.88 |  | Difficulty hearing | 0.76 |  |
|  |  |  | Tense | 0.78 |  | Difficulty remembering | 0.62 |  |
|  |  |  | Depressed | 0.76 |  |  | α = 0.62 |  |
|  |  |  | Irritable | 0.76 |  |  |  |  |
|  |  |  | Constipation | 0.75 |  |  |  |  |
|  |  |  | Distinguishing hot-cold | 0.51 |  |  |  |  |
|  |  |  |  | α = 0.90 |  |  |  |  |
|  |  |  |  |  |  |  |  |  |
| **Standing/walking feeling ground** | 0.98 | 4.88 | **Standing/walking feeling ground** | 0.53 | 6.62 | **Standing/walking feeling ground** | 0.61 | 21.51 |
| Difficulty remembering | 0.98 |  | Walking feet drop | 0.76 |  | Walking feet drop | 0.97 |  |
| Nausea | 0.86 |  |  | α = 0.52 |  | Holding pen | 0.97 |  |
| Difficulty concentration | 0.78 |  |  |  |  | Opening jar | 0.87 |  |
| Diarrhea | 0.66 |  |  |  |  | Burning pain in hands | 0.83 |  |
| Climbing stairs | 0.60 |  |  |  |  | Climbing stairs | 0.76 |  |
|  | α = 0.72 |  |  |  |  | Dizzy | 0.69 |  |
| Walking feet drop | 0.93 | 8.08 |  |  |  | Cramps in hands | 0.61 |  |
| Distinguishing hot-cold | 0.93 |  |  |  |  | Constipation | 0.56 |  |
|  | α = 1.00 |  |  |  |  |  | α = 0.78 |  |
|  |  |  |  |  |  |  |  |  |
| Cramps in feet | 0.97 | 1.76 | Cramps in feet | 0.86 | 13.98 | Cramps in feet | 0.54 | 13.56 |
| Irritable | 0.97 |  | Burning pain in hands | 0.87 |  | Vomiting | 0.90 |  |
|  | α = 1.00 |  | Cramps in hands | 0.81 |  | Appetite loss | 0.82 |  |
|  |  |  | Holding pen | 0.60 |  | Diarrhea | 0.79 |  |
|  |  |  | Burning pain in feet | 0.58 |  | Trouble sleeping | 0.63 |  |
|  |  |  | Difficulty remembering | 0.56 |  |  | α = 0.83 |  |
|  |  |  | Dizzy | 0.56 |  |  |  |  |
|  |  |  |  | α = 0.85 |  |  |  |  |
|  |  |  |  |  |  |  |  |  |
| Constipation | 0.81 | 5.56 | Difficulty concentration | 0.74 | 8.81 |  |  |  |
| Difficulty hearing | 0.60 |  | Short of breath | 0.71 |  |  |  |  |
| Short of breath | 0.46 |  | Climbing stairs | 0.64 |  |  |  |  |
|  | α = 0.64 |  | Difficulty hearing | 0.50 |  |  |  |  |
|  |  |  | Diarrhea | -0.50 |  |  |  |  |
|  |  |  |  | α = 0.68 |  |  |  |  |
|  |  |  |  |  |  |  |  |  |
| Vomiting | 0.77 | 5.27 |  |  |  |  |  |  |
| Numbness in hands | 0.61 |  |  |  |  |  |  |  |
|  | α = 0.67 |  |  |  |  |  |  |  |
|  |  |  |  |  |  |  |  |  |
| Appetite loss | 0.69 | 4.47 |  |  |  |  |  |  |
| Opening jar | 0.60 |  |  |  |  |  |  |  |
|  | α = 0.41 |  |  |  |  |  |  |  |
