# Supplementary material for: Redefining chemotherapy-induced peripheral neuropathy through symptom cluster analysis and patient-reported outcome data over time
Source: BMC Cancer. 2019 Nov 27;19:1151. doi: 10.1186/s12885-019-6352-3 (PMC6882224; doi:10.1186/s12885-019-6352-3)
Supplement: Supplementary file 4 — Additional file 4: Table S4. Influencing factors for the sensory neuropathy symptom cluster over time. Table S5. Influencing factors for the motor-sensory neuropathy symptom cluster over time. Table S6. Influencing factors for the sensorimotor neuropathy symptom cluster over time. Table S7. Influencing factors for the autonomic neuropathy symptom cluster over time. [file 12885_2019_6352_MOESM4_ESM.docx]

**Table S4** Influence factors for sensory neuropathy symptom cluster over time

| Coefficients | | | | | | |
| --- | --- | --- | --- | --- | --- | --- |
| Time points | | Unstandardized coefficients | | Standardized coefficients | t | Sig. |
|  |  | B | Std. error | Beta |  |  |
| T1 | - | - | - | - | - | - |
| T2 | Caucasian | 4.337 | 1.724 | .358 | 2.515 | .016 |
| T3 | Stage IV cancer | 3.139 | 1.081 | .381 | 2.905 | .006 |
|  | Non-Chinese Asian | 4.165 | 1.636 | .334 | 2.546 | .015 |
| T4 | Non-Chinese Asian | 4.497 | 1.471 | .397 | 3.058 | .004 |
|  | Stage IV cancer | 2.634 | .974 | .350 | 2.704 | .010 |
|  | Neoadjuvant chemotherapy | 3.497 | 1.471 | .309 | 2.378 | .022 |
| T5 | Non-Chinese Asian | 6.407 | 1.165 | .616 | 5.499 | .000 |
|  | Gender | 2.622 | .755 | .396 | 3.473 | .001 |
|  | Adjuvant chemotherapy | -1.806 | .755 | -.273 | -2.392 | .022 |
| T6 | Non-Chinese Asian | 10.368 | 2.906 | .644 | 3.568 | .002 |
| T7 | Stage IV cancer | 3.364 | 1.116 | .614 | 3.014 | .009 |
| T8 | - | - | - | - | - | - |
| T9 | - | - | - | - | - | - |
| T10 | Non-Chinese Asian | 10.682 | 1.267 | .874 | 8.429 | .000 |
| a. Dependent variable: sensory neuropathy symptom cluster | | | | | | |

**Table S5** Influence factors for motor-sensory neuropathy symptom cluster over time

| Coefficients | | | | | | |
| --- | --- | --- | --- | --- | --- | --- |
| Time points | | Unstandardized coefficients | | Standardized coefficients | t | Sig. |
|  |  | B | Std. error | Beta |  |  |
| T1 | Non-Chinese Asian | 1.083 | .244 | .524 | 4.441 | .000 |
| T2 | Caucasian | .974 | .278 | .444 | 3.501 | .001 |
|  | Non-Chinese Asian | .574 | .182 | .399 | 3.145 | .003 |
| T3 | Non-Chinese Asian | 2.960 | .969 | .376 | 3.055 | .004 |
|  | Caucasian | 5.175 | 1.405 | .476 | 3.685 | .001 |
|  | Age | -.097 | .040 | -.312 | -2.411 | .020 |
| T4 | - | - | - | - | - | - |
| T5 | Non-Chinese Asian | 3.662 | .494 | .777 | 7.407 | .000 |
| T6 | Neoadjuvant chemotherapy | 1.500 | .373 | .688 | 4.025 | .001 |
| T7 | - | - | - | - | - | - |
| T8 | Caucasian | 6.244 | 1.755 | .478 | 3.559 | .001 |
|  | Non-Chinese Asian | 3.868 | 1.446 | .358 | 2.675 | .012 |
|  | Gender | 2.259 | .866 | .350 | 2.608 | .014 |
| T9 | Caucasian | 1.462 | .469 | .479 | 3.113 | .004 |
|  | Non-Chinese Asian | .962 | .390 | .379 | 2.465 | .020 |
| T10 | - | - | - | - | - | - |
| a. Dependent variable: motor-sensory neuropathy symptom cluster | | | | | | |

**Table S6** Influence factors for sensorimotor neuropathy symptom cluster over time

| Coefficients | | | | | | |
| --- | --- | --- | --- | --- | --- | --- |
| Time points | | Unstandardized coefficients | | Standardized coefficients | t | Sig. |
|  |  | B | Std. error | Beta |  |  |
| T1 | Caucasian | 287.702 | 63.465 | .560 | 4.533 | .000 |
|  | Age | -4.697 | 2.336 | -.249 | -2.010 | .050 |
| T2 | Caucasian | 3.217 | .788 | .530 | 4.080 | .000 |
|  | Age | -.065 | .023 | -.372 | -2.869 | .006 |
|  | Non-Chinese Asian | 1.346 | .491 | .338 | 2.743 | .009 |
| T3 | Caucasian | 2.659 | .885 | .413 | 3.006 | .004 |
| T4 | Caucasian | 7.695 | 2.270 | .468 | 3.390 | .002 |
| T5 | - | - | - | - | - | - |
| T6 | Non-Chinese Asian | 6.368 | 2.248 | .555 | 2.833 | .011 |
| T7 | - | - | - | - | - | - |
| T8 | Stage IV cancer | 4.875 | 1.611 | .448 | 3.027 | .005 |
|  | Non-Chinese Asian | 5.667 | 2.429 | .346 | 2.333 | .026 |
| T9 | Caucasian | 3.621 | 1.764 | .356 | 2.053 | .049 |
| T10 | Non-Chinese Asian | 5.955 | 1.558 | .632 | 3.821 | .001 |
| a. Dependent variable: sensorimotor neuropathy symptom cluster | | | | | | |

**Table S7** Influence factors for autonomic neuropathy symptom cluster over time

| Coefficients | | | | | | |
| --- | --- | --- | --- | --- | --- | --- |
| Time points | | Unstandardized coefficients | | Standardized coefficients | t | Sig. |
|  |  | B | Std. error | Beta |  |  |
| T1 | Caucasian | 248.910 | 62.262 | .485 | 3.998 | .000 |
| T2 | Caucasian | 10.942 | 3.092 | .480 | 3.539 | .001 |
|  | Non-Chinese Asian | 5.202 | 1.924 | .348 | 2.703 | .010 |
|  | Age | -.196 | .088 | -.301 | -2.223 | .032 |
| T3 | Non-Chinese Asian | 1.417 | .387 | .483 | 3.659 | .001 |
| T4 | Caucasian | 1.817 | .795 | .336 | 2.286 | .027 |
| T5 | Non-Chinese Asian | 1.882 | .550 | .495 | 3.420 | .002 |
| T6 | - | - | - | - | - | - |
| T7 | Non-Chinese Asian | 5.167 | 2.018 | .551 | 2.560 | .022 |
| T8 | Caucasian | 3.292 | 1.219 | .397 | 2.699 | .011 |
|  | Gender | 1.561 | .603 | .381 | 2.587 | .014 |
| T9 | Neoadjuvant chemotherapy | 4.155 | 1.095 | .576 | 3.795 | .001 |
| T10 | Non-Chinese Asian | 2.103 | .411 | .672 | 5.122 | .000 |
|  | Stage III cancer | .681 | .241 | .371 | 2.829 | .010 |
| a. Dependent variable: autonomic neuropathy symptom cluster | | | | | | |
